# Supplementary material for: Curated and harmonised transcriptomics datasets of interstitial lung diseases
Source: Data Brief. 2025 Oct 14;63:112139. doi: 10.1016/j.dib.2025.112139 (PMC12581653; doi:10.1016/j.dib.2025.112139)
Supplement: Supplementary file 1 [file mmc1.zip › Supplementary_material/RNA-seq/fastQC reports/GSE169500/TRIMMED_SRR14057106_fastqc.html]

TRIMMED\_SRR14057106.fastq.gz FastQC Report 

FastQC Report

Sat 3 Dec 2022  
TRIMMED\_SRR14057106.fastq.gz

## Summary

- Basic Statistics
- Per base sequence quality
- Per sequence quality scores
- Per base sequence content
- Per sequence GC content
- Per base N content
- Sequence Length Distribution
- Sequence Duplication Levels
- Overrepresented sequences
- Adapter Content

## Basic Statistics

| Measure | Value |
| --- | --- |
| Filename | TRIMMED\_SRR14057106.fastq.gz |
| File type | Conventional base calls |
| Encoding | Sanger / Illumina 1.9 |
| Total Sequences | 16367646 |
| Sequences flagged as poor quality | 0 |
| Sequence length | 35-371 |
| %GC | 49 |

## Per base sequence quality

## Per sequence quality scores

## Per base sequence content

## Per sequence GC content

## Per base N content

## Sequence Length Distribution

## Sequence Duplication Levels

## Overrepresented sequences

| Sequence | Count | Percentage | Possible Source |
| --- | --- | --- | --- |
| GGGCCACGAGCTGAGTGCGTCCTGTCACTCCACTCCCATGTCCCTTGGGA | 200469 | 1.224788219393308 | No Hit |
| GCTGCTACAGGAGAATAGCAGACAGGTATAGTTAAGAAACAACTTTATTT | 106703 | 0.6519141481921102 | No Hit |
| ACACTGAGGACTCTGTTCCTCTCCTTTCCGCCTAGGGGAAAGTCCCCGGA | 67389 | 0.4117207813512096 | No Hit |
| AAAGCCAAGAAAACCAACGATGCCAGATACTGAATTTCTATAGCACTGAC | 65533 | 0.4003813376706705 | No Hit |
| GGGAATGTCTACGTGCGTATGCACGTGGCACTCTCT | 60262 | 0.3681775620025018 | No Hit |
| TCTTTCTGGCCTGGAGGCTATCCAGCGTACTCCAAAGATTCAGGTTTACT | 48145 | 0.2941473685342413 | No Hit |
| GGGAATGTCTACGTGCGTATGCACGTGGCACTCTCTGC | 37252 | 0.2275953426656466 | No Hit |
| TCTGCAATGCAATCACAATGCCCAAACTAGACCTGCCATTTCTCACACAC | 35089 | 0.2143802474711391 | No Hit |
| CTAAGTGCTGTGTTGTCGTTCCCCCTGCTTAAAATAAAGTTGTTTCTTAA | 31633 | 0.19326542130737676 | No Hit |
| GGGAATGTCTACGTGCGTATGCACGTGGCACTCTCTGCC | 31156 | 0.1903511354045658 | No Hit |
| TCTGTCTTCTTCAGTTTCGACTTATCGAATTTCTCGATCTCAGCCATATC | 28853 | 0.17628069424277626 | No Hit |
| GGGCCACGAGCTGAGTGCGTCCTGTCACTCCACTCCCATGTCCCTTGGGAAGGTCTGAGACTAGGGCCAGAGGCG | 28740 | 0.17559030785489863 | No Hit |
| TCACACTTCATGATGGAGTTGAAGGTAGTTTCGTGGATGCCACAGGACTC | 28650 | 0.1750404425902173 | No Hit |
| AAGACCAACGTCAAGGCCGCCTGGGGTAAGGTCGGCGCGCACGCTGGCGA | 27783 | 0.1697434072071207 | No Hit |
| AAGCTCAGGGAGAGCCTGTTAGGGCCGCCTCTGGCCTAGTCTCAGACCTT | 27608 | 0.16867422474801813 | No Hit |
| ACATAGCAATTCAGGAAATTTGACTTTCCATTCTCTGCTGGATGACGTGA | 27402 | 0.16741564425330313 | No Hit |
| AAGCTCAGGGAGAGCCTGTTAGGGCCGCCTCTGGCCCTAGTCTCAGACCT | 26361 | 0.161055536025156 | No Hit |
| GGGAATGTCTACGTGCGTATGCACGTGGCACTCTCTGCCGAGGTCCGGG | 25160 | 0.15371788954868648 | No Hit |
| TGTTCCCTCTCCTCATGAGATTGGTGAAGAAAGTATTTGGCAAAGTTCTT | 24865 | 0.15191555340334217 | No Hit |
| TACTCGTGCGCCTCGCTTTGCTTTTCCTCCGCAACCATGTCTGACAAACC | 24609 | 0.15035149220602645 | No Hit |
| ATTCTTCCTACCCATGAGCATGGAATGTTCTTCCATTTGTTTGTATCCTC | 23646 | 0.14446793387393642 | No Hit |
| GAAGACGTACCTTTGTTGGAAGACATACCTTTGTTGGAAGACGTACCTTT | 21343 | 0.13039749271214687 | No Hit |
| TGACTGGTGATGCTGATATTGGAGTGCATCTGAGGCTGTTGTAGAAATAA | 21063 | 0.1286868007775828 | No Hit |
| TTTACACCTGCTAGGGTGTTCAAAGGTCAGTGCTATAGAAATTCAGTATC | 20618 | 0.1259680225244363 | No Hit |
| TGTCCATGTCTTACTACTTTGACCGCGATGATGTGGCTTTGAAGAACTTT | 20585 | 0.12576640526071986 | No Hit |
| TGGCTCTCCCAGAGGCAGAAGGACAATGGCTGTTTCAGGAGCTCTGGGTC | 20354 | 0.12435508441470447 | No Hit |
| GGGAATGTCTACGTGCGTATGCACGTGGCACTCTCTGCCCGAGGTC | 20196 | 0.12338976539448616 | No Hit |
| GGGAATGTCTACGTGCGTATGCACGTGGCACTCTCTGCCCGAGGTCCGGG | 20109 | 0.1228582289719609 | No Hit |
| AGCGGTTCCGCTGCCCTGAGGCACTCTTCCAGCCTTCCTTCCTGGGCATG | 19679 | 0.12023109492959463 | No Hit |
| CAAGTATGTGAAACAAGAAGTTCTGGGTCCTTTCATCATAAGGGAGAAGC | 19236 | 0.1175245359045522 | No Hit |
| AAAACAGCGTCTTACTGGGGAGGGATATGTGCTTTCTCATCTATCCTCAG | 18945 | 0.11574663821541595 | No Hit |
| TGTTGTGCTAAACGCTGGGAGGGTACAAGTTGGTCATTCCTAAATCTGTG | 18786 | 0.11477520958114563 | No Hit |
| AAACCAGACATGGGGGAAATCGCCAGCTTCGATAAGGCCAAGCTGAAGAA | 17839 | 0.10898940507388785 | No Hit |
| GGTTATAATCATTGGCAATGTTGTAATCAACAACCCATGTTATTTCTACA | 17081 | 0.10435831762246081 | No Hit |

## Adapter Content

Produced by FastQC (version 0.11.7)
